# TNFAIP3 Deficiency Affects Monocytes, Monocytes-Derived Cells and Microglia in Mice

**DOI:** 10.3390/ijms21082830

**Published:** 2020-04-18

**Authors:** Francesca Montarolo, Simona Perga, Carlotta Tessarolo, Michela Spadaro, Serena Martire, Antonio Bertolotto

**Affiliations:** 1 Neuroscience Institute Cavalieri Ottolenghi (NICO), Orbassano, 10043 Turin, Italy; simona.perga@unito.it (S.P.); carlotta.tessarolo@gmail.com (C.T.); spadaro_michela@yahoo.it (M.S.); serena.martire@gmail.com (S.M.); antonio.bertolotto@gmail.com (A.B.); 2 Neurobiology Unit, Neurology–CReSM (Regional Referring Center of Multiple Sclerosis), AOU San Luigi Gonzaga, Orbassano, 10043 Turin, Italy; 3 Department of Molecular Biotechnology and Health Sciences, University of Turin, 10126 Turin, Italy; 4 Department of Neuroscience “Rita Levi Montalcini”, University of Turin, 10125 Turin, Italy

**Keywords:** TNFAIP3, inflammation, myeloid cells, microglia, monocyte and macrophage

## Abstract

The intracellular-ubiquitin-ending-enzyme tumor necrosis factor alpha-induced protein 3 (TNFAIP3) is a potent inhibitor of the pro-inflammatory nuclear factor kappa-light-chain-enhancer of activated B cell (NF-kB) pathway. Single nucleotide polymorphisms in TNFAIP3 locus have been associated to autoimmune inflammatory disorders, including Multiple Sclerosis (MS). Previously, we reported a TNFAIP3 down-regulated gene expression level in blood and specifically in monocytes obtained from treatment-naïve MS patients compared to healthy controls (HC). Myeloid cells exert a key role in the pathogenesis of MS. Here we evaluated the effect of specific TNFAIP3 deficiency in myeloid cells including monocytes, monocyte-derived cells (M-MDC) and microglia analyzing lymphoid organs and microglia of mice. TNFAIP3 deletion is induced using conditional knock-out mice for myeloid lineage. Flow-cytometry and histological procedures were applied to assess the immune cell populations of spleen, lymph nodes and bone marrow and microglial cell density in the central nervous system (CNS), respectively. We found that TNFAIP3 deletion in myeloid cells induces a reduction in body weight, a decrease in the number of M-MDC and of common monocyte and granulocyte precursor cells (CMGPs). We also reported that the lack of TNFAIP3 in myeloid cells induces an increase in microglial cell density. The results suggest that TNFAIP3 in myeloid cells critically controls the development of M-MDC in lymphoid organ and of microglia in the CNS.

## 1. Introduction

Inflammation is an adaptive physiological response to cell injury characterized by the production of pro- and anti-inflammatory mediators that establish both innate and acquired immune response [1]. During physiological conditions, inflammation restores tissue integrity and function. However, in case of prolonged or non-resolving inflammatory events, inflammation can evolve into detrimental process contributing to the pathogenesis of chronic inflammatory disorders, such as autoimmune diseases. Many signaling molecules produced by inflammatory events are under the control of the transcription factor nuclear factor kappa-light-chain-enhancer of activated B cells (NF-kB), able to induce inflammatory responses through the activation and the production of pro-inflammatory cytokines, cellular mediators and response genes [2]. In this context, the intracellular-ubiquitin-ending-enzyme tumor necrosis factor alpha-induced protein 3 (TNFAIP3) is one of the most potent inhibitor of the NF-kB pathway [3]. TNFAIP3 is a cytoplasmic zinc finger protein originally identified as a TNF-inducible protein. The biochemical mechanisms by which TNFAIP3 restricts NF-κB signaling are complex [4]. TNFAIP3 has a deubiquitinating N-terminus domain and an ubiquitin ligase C-terminus domain that cooperate to inhibit the NF-kB pathway [5]. Hence, TNFAIP3 appears to be a dual function enzyme that adds and subtracts ubiquitin moieties to deactivate and degrade several NF-kB signaling molecules, such as the receptor interacting protein-1 (RIP1) and TNF receptor-associated factor (TRAF) [6].

Single nucleotide polymorphisms (SNPs) in the TNFAIP3 locus have been associated to autoimmune disorders such as systemic lupus erythematous (SLE) [7], rheumatoid arthritis (RA) [7,8], psoriasis [9], type one diabetes [10], coeliac disease [10] and multiple sclerosis (MS) [11]. Previously, we reported a decrease in gene expression level of TNFAIP3 in whole blood and in peripheral blood mononuclear cells (PBMCs) obtained from treatment naïve MS patients in comparison to healthy controls (HC) [12,13,14,15]. Remarkably, we observed the abnormal expression levels of TNFAIP3 being reverted in MS patients during pregnancy, which represents a transitory state of immune tolerance associated with reduced disease activity [12]. We also demonstrated a negative correlation between the TNFAIP3 gene expression level and the clinical parameters such as the relapse rate and Expanded Disability Status Scale (EDSS) score, indicating that the most aggressive forms of MS are characterized by lower level of TNFAIP3 expression [13]. Specifically, we found the down-regulation of the TNFAIP3 gene expression level in CD14+ monocytes obtained from MS patients compared to HC [16]. 

Different studies highlighted the important role of myeloid cells which include ontogenetically distinct cell populations, such as microglia, monocytes and monocytes-derived cells (M-MDC) such as macrophages and dendritic cells (DCs) in the pathogenic autoimmune process of MS. Indeed, activated macrophages and microglia are hallmarks of active lesions with ongoing demyelination and axonal injury in MS able to induce neuro-inflammation in the brain parenchyma [17]. However, although excessively activated myeloid cells contribute to MS pathology, aspects of myeloid cell function, such as their production of anti-inflammatory and neurotrophic factors, their removal of myelin and cellular debris and their protection of neurons, are beneficial to restore tissue integrity [18].

To date, the specific evaluation of the effects of the TNFAIP3 deficiency in monocytes arises from few studies with knock-out (KO) murine models. Mice full deficient in TNFAIP3 develop severe systemic inflammation, hypersensitive to both lipopolysaccharide and TNF-α, cachexia and premature death [19]. In contrast, the circulating hematopoietic- [19,20,21,22,23,24] and specific myeloid- [22] TNFAIP3 deficient mice do not die prematurely but develop similar inflammatory signs. Interestingly, the myeloid-TNFAIP3 deficient mice show spontaneous polyarthritis and high levels of inflammatory cytokines in their serum, consistent with a sustained NF-kB activation [20]. Notably, the in vitro cultured TNFAIP3-deficient macrophages produce an increased amount of the inflammatory cytokines such as interleukin (IL)-1β, IL-6, IL-18, TNF-α, interferon (IFN), chemokine (C–X–C motif) ligand (CXCL)9 and CXCL10 [20,21], indicating that the lack of TNFAIP3 alters the functionality of myeloid cells. Recently, Voet and colleagues investigated the role of TNFAIP3 in microglia using a targeting strategy based on the longevity and capacity of self-renewal of myeloid cells [22]. Mice lacking the TNFAIP3 gene in microglia showed an increased microglial cell density and an altered neuronal synaptic function in cortical circuits [22]. The authors also demonstrated that microglia-specific TNFAIP3 deficiency exacerbates the course of the immune-mediated mouse model of MS, represented by the Experimental Autoimmune Encephalomyelitis (EAE). However, the analysis of the effects of TNFAIP3 deficiency in the myeloid cells including ontogenetically distinct cell populations, such as M-MDC and microglia is still lacking. Hence, we evaluated the effect of the simultaneously TNFAIP3 deficiency in M-MDC and microglia in lymphoid organs and in the central nervous system (CNS) parenchyma.

## 2. Results

**Figure 1:**
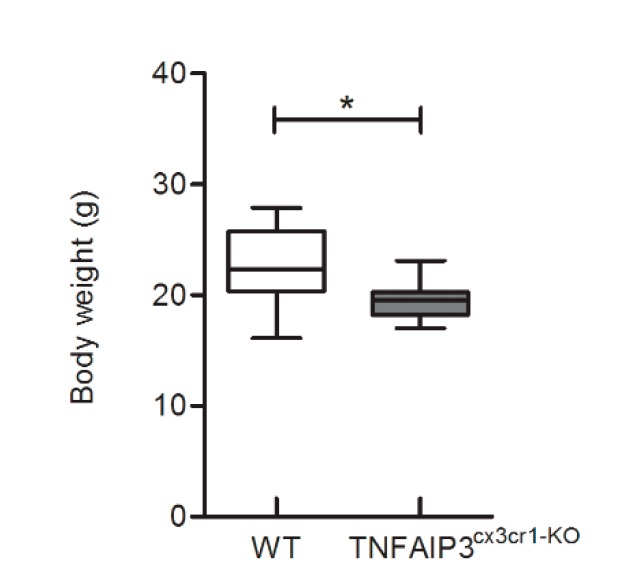
Body weight analysis of 3 month-old WT and TNFAIP3^cx3cr1-KO^ mice. The analysis of body weight reports that at 3 months of age, TNFAIP3^cx3cr1-KO^ mice are smaller compared to their WT littermates (*n* = 7 for each group) (Mann–Whitney test, *p* = 0.014 * *p* < 0.05).

To characterize the phenotype of adult TNFAIP3^cx3cr1-KO^ mice, their body weight was recorded at 3 months of age. TNFAIP3^cx3cr1-KO^ mice (*n* = 7) showed a lower body weight compared to their WT littermates (*n* = 7) (Figure 1, Mann–Whitney test *p* = 0.014). No others macroscopic abnormalities were observed in TNFAIP3^cx3cr1-KO^ mice in comparison to their WT littermates.

**Figure 2:**
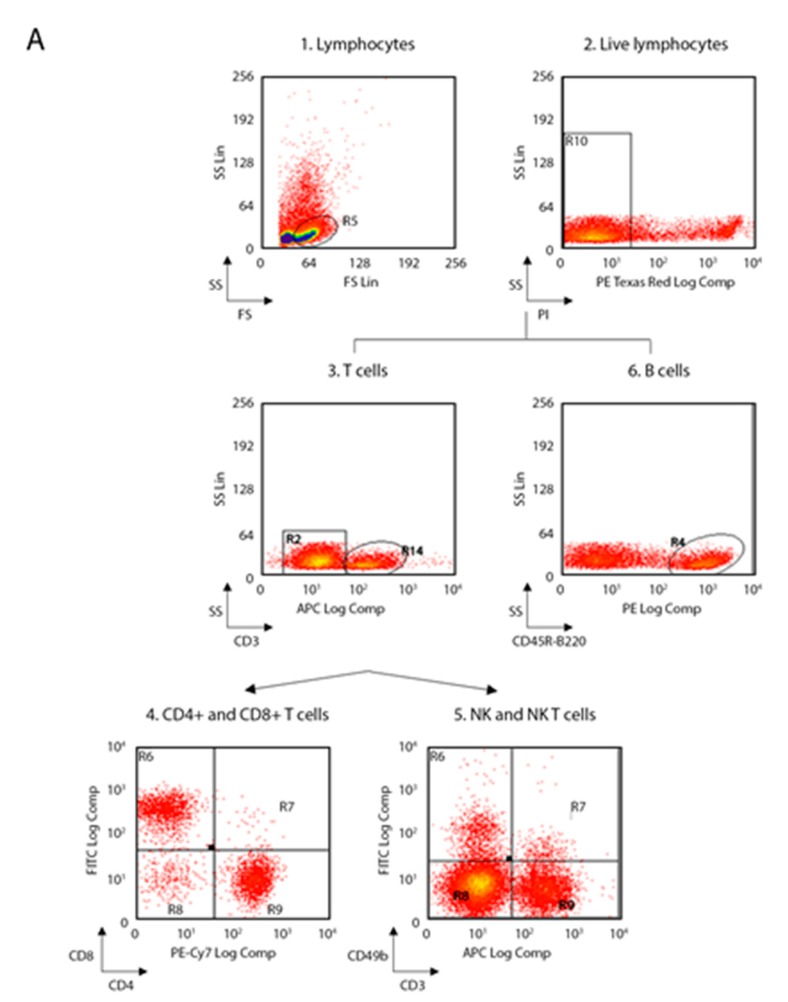

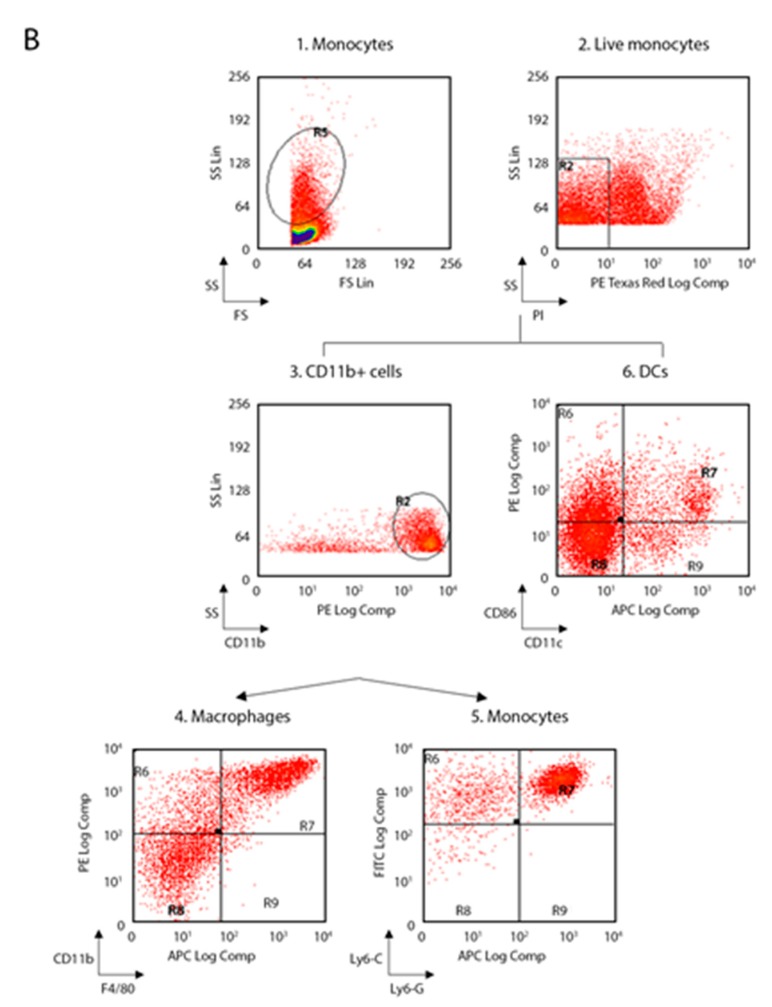
Flow-cytometry gating strategy used for spleen and lymph nodes. (**A**) Gating strategy for lymphocytes, NK and B cells; (1) FS versus SS gating was used to discriminate cells based on their size; (2) living cells were then identified by propidium iodide negativity; (3) CD3 was used as a T-cell marker; (4) CD3+ cells were distinguished based on the presence of CD4 and CD8; (5) NK and NK T were distinguished based on the presence of CD49b; (6) CD45R was used as a B-cell marker. (**B**) Gating strategy for macrophages, monocytes and DCs; (1) FS versus SS gating was used to discriminate cells based on their size; (2) living cells were then identified by propidium iodide negativity; (3–4) F4/80 gating on CD11b+ cells was used to select macrophages; (5) Ly6-C and Ly6-G gating on CD11b+ cells was used to select monocytes; (6) DCs were distinguished based on the presence of CD11c and CD86. (NK, natural killer; FS, forward scatter; SS side scatter; DCs, dendritic cells).

**Figure 3:**
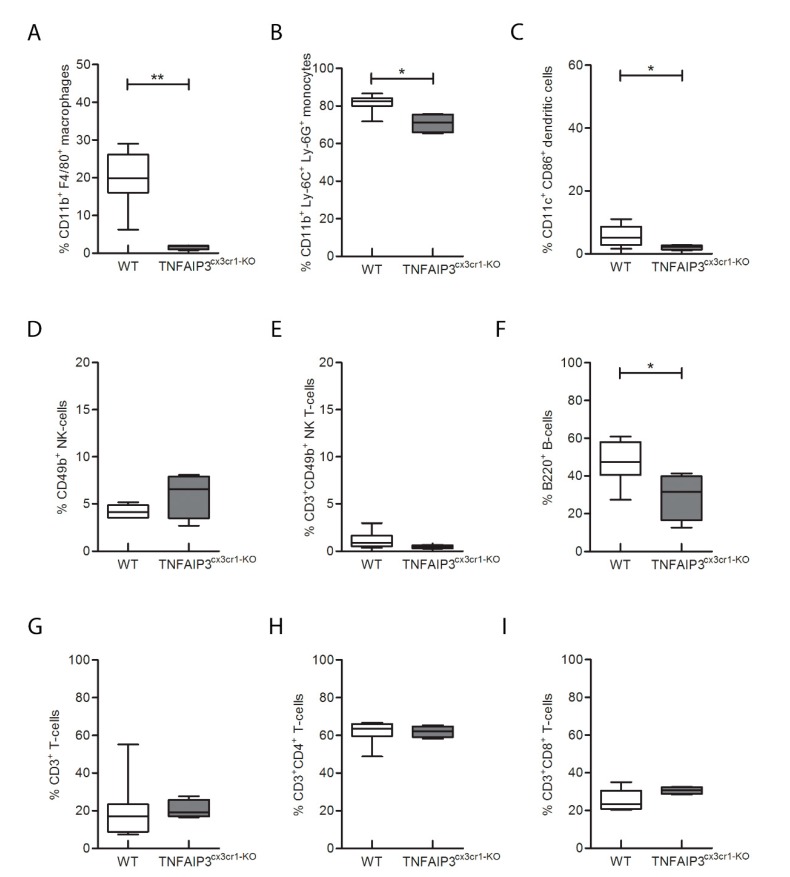
Flow-cytometry analysis performed on spleen obtained from 3 month-old WT and TNFAIP3^cx3cr1-KO^ mice. The analysis reveals that the percentage number of CD11b^+^F4/80^+^ macrophages (**A**, *p* = 0.004), CD11b^+^Ly-6C^+^Ly-6G^+^ monocytes (**B**, *p* = 0.016), CD11c^+^CD86^+^ DCs (**C**, *p* = 0.048) and B200^+^ B (**F**, *p* = 0.048) cells is reduced in TNFAIP3^cx3cr1-KO^ mice compared to their WT littermates. No differences are reported in CD49^+^NK (**D**, *p* = 0.214) and CD3^+^ CD49^+^NK T cells (**E**, *p* = 0.153) CD3^+^ T (**G**, *p* = 0.367), CD3^+^CD4^+^ T helper (**H**, *p* = 0.683) and CD3^+^CD8^+^ cytotoxic T cells (**I**, *p* = 0.109) (WT *n* = 8; TNFAIP3^cx3cr1-KO^ *n* = 4) (Mann–Whitney test, ** *p* < 0.01; * *p* < 0.05). (DCs, dendritic cells; NK, natural killer).

**Figure 4:**
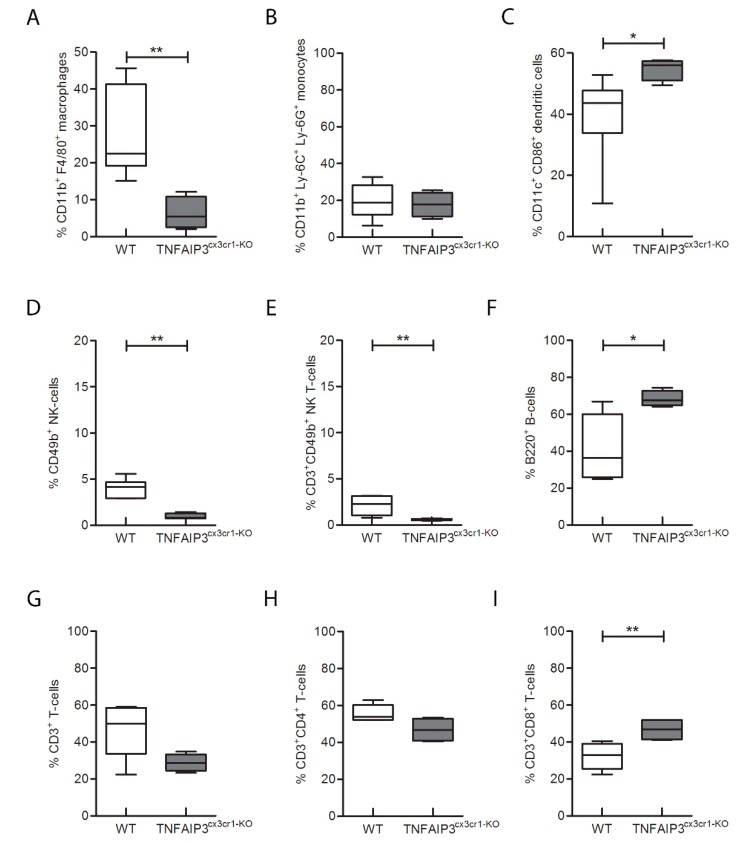
Flow-cytometry analysis performed on lymph nodes obtained from 3 month-old WT and TNFAIP3^cx3cr1-KO^ mice. The analysis reveals that the percentage number of CD11b^+^F4/80^+^ macrophages (**A**, *p* = 0.006), CD49^+^ NK (**D**, *p* = 0.009) and CD3^+^CD49^+^ NK T (**E**, *p* = 0.009) cells is reduced in TNFAIP3^cx3cr1-KO^ mice compared to their WT littermates. No differences are reported in CD11b^+^Ly-6C^+^Ly-6G^+^ monocytes (**B**, *p* = 0.914), CD3^+^ T (**G**, *p* = 0.114) and CD3^+^CD4^+^ T helper cells (**H**, *p* = 0.066). The percentage number of CD11c^+^CD86^+^ DCs (**C**, *p* = 0.019), B200^+^ B (**F**, *p* = 0.019) and CD3^+^CD8^+^ cytotoxic T (**I**, *p* = 0.009) cells is increased in TNFAIP3^cx3cr1-KO^ mice compared to their WT littermates. (WT *n* = 8; TNFAIP3^cx3cr1-KO^ *n* = 4) (Mann–Whitney test, ** *p* < 0.01; * *p* < 0.05). (DCs, dendritic cells; NK, natural killer).

To characterize the immunological phenotype of TNFAIP3^cx3cr1-KO^ mice due to the absence of TNFAIP3 gene, the flow-cytometry analysis on samples obtained from spleen (Figure 2 and Figure 3 and Table S1) and lymph nodes (Figure 2 and Figure 4 and Table S1) of adult 3 month old WT (*n* = 8) and TNFAIP3^cx3cr1-KO^ (*n* = 4) mice was performed. Notably, the analysis on the spleens highlighted that the percentage number of macrophages (Figure 3A, Mann–Whitney test *p* = 0.004), monocytes (Figure 3B, Mann–Whitney test *p* = 0.016), DCs (Figure 3C, Mann–Whitney test *p* = 0.048) and B cells (Figure 3F, Mann–Whitney test *p* = 0.048) was reduced in TNFAIP3^cx3cr1-KO^ mice compared to their WT littermates. No differences were reported in the other cellular populations analyzed, such as natural killer (NK) (Figure 3D, Mann–Whitney test *p* = 0.214), NK T (Figure 3E, Mann–Whitney test *p* = 0.153), CD3+ T lymphocytes (Figure 3G, Mann–Whitney test *p* = 0.367), CD4+ T helper (Figure 3H, Mann–Whitney test *p* = 0.683) and CD8+ T cytotoxic (Figure 3I, Mann–Whitney test *p* = 0.109) cells. These results indicate that the deletion of TNFAIP3 in myeloid cells leads to an altered immunological phenotype not only in myeloid linage as expected but also in lymphocytes, such as B cells.

In order to corroborate the results obtained on spleen, the immunological phenotype analysis of lymph nodes obtained from the same mice was performed (Figure 4; WT *n* = 8, TNFAIP3^cx3cr1-KO^ *n* = 4). TNFAIP3^cx3cr1-KO^ showed a reduction of the percentage number of macrophages (Figure 4A, Mann–Whitney test *p* = 0.006), NK (Figure 4D, Mann–Whitney test *p* = 0.009) and NK T (Figure 4E, Mann–Whitney test *p* = 0.009) cells in comparison to their WT littermates. Interestingly, the percentage number of monocytes was not altered in TNFAIP3^cx3cr1-KO^ mice (Figure 4B, Mann–Whitney test *p* = 0.914). Conversely, the percentage number of DCs (Figure 4C Mann–Whitney test *p* = 0.019), B (Figure 4F, Mann–Whitney test *p* = 0.019) and CD8+ T cytotoxic (Figure 4I, Mann–Whitney test *p* = 0.009) cells was increased in TNFAIP3^cx3cr1-KO^ mice in comparison to WT littermates. No differences emerged in the percentage number of CD3+ T (Figure 4G Mann–Whitney test *p* = 0.114) and CD4+ T helper (Figure 4H Mann–Whitney test *p* = 0.066) cells in TNFAIP3^cx3cr1-KO^ mice in comparison to their WT littermates. Collectively, these results indicate that the lack of TNFAIP3 in myeloid cells impairs also the immunological phenotype at level of lymph nodes.

**Figure 5:**
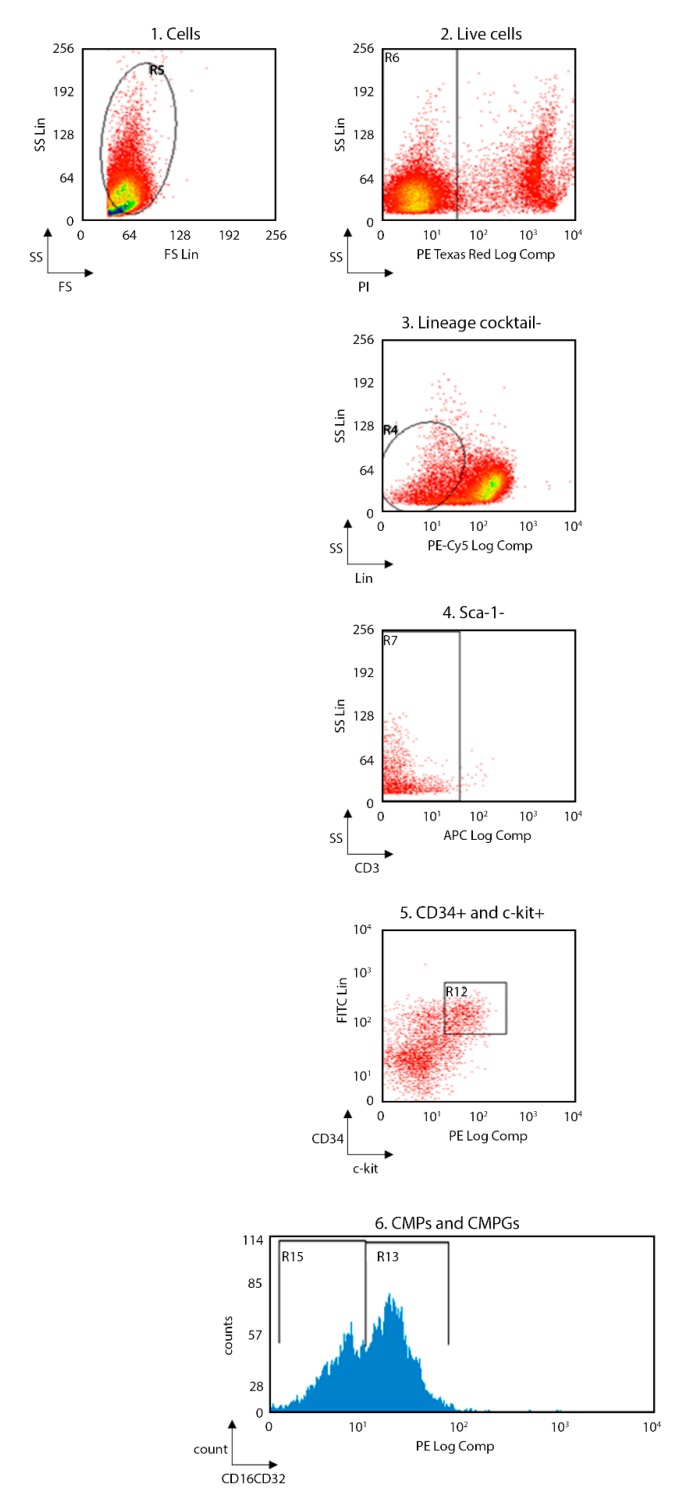
Flow-cytometry gating strategy used for bone marrow. Gating strategy for CMPs and CMGPs; (1) FS versus SS gating was used to discriminate cells based on their size; (2) living cells were then identified by propidium iodide negativity; (3) Lin- and (4) Sca-1- cells were gated based on the presence of (5) CD34 and c-kit; (6) CMPs and CMGPs were distinguished based on the presence of CD16CD32. (CMPs, common myeloid precursors; CMGPs, common monocyte and granulocyte precursor; FS, forward scatter; SS side scatter).

**Figure 6:**
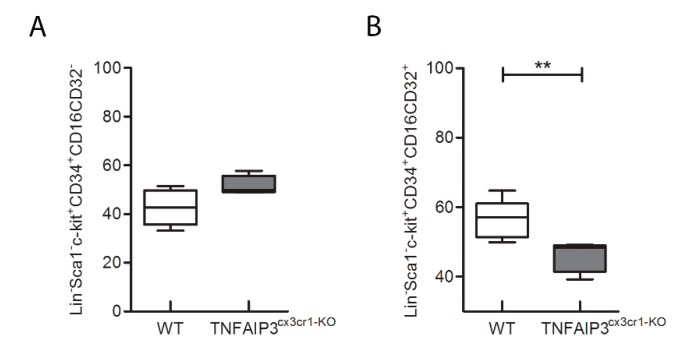
Flow-cytometry analysis performed on bone marrow obtained from 3 month-old WT and TNFAIP3^cx3cr1-KO^ mice. No differences are highlighted between WT and TNFAIP3^cx3cr1-KO^ mice in the percentage number of the Lin^-^Sca1^-^c-kit^+^CD34^+^CD16CD32^+^ CMPs (**A**, *p* = 0.073). However, the percentage number of the CMGPs is reduced in TNFAIP3^cx3cr1-KO^ mice compared to their WT littermates (**B**, *p* = 0.006). (WT *n* = 8; TNFAIP3^cx3cr1-KO^ *n* = 4) (Mann–Whitney test, ** *p* < 0.01). (CMPs, common myeloid precursors; CMGPs, common monocyte and granulocyte precursor).

In order to evaluate whether the immunological alteration occurs during the development of immune cells or it is already present in the precursor cells, the flow-cytometry analysis was performed also in bone marrow of the same TNFAIP3^cx3cr1-KO^ and WT mice (Figure 5 and Figure 6; WT *n* = 8, TNFAIP3^cx3cr1-KO^ *n* = 4). Specifically, we investigated the consequences of TNFAIP3 ablation in the common myeloid precursors (CMPs, Figure 6A) and in the common monocyte and granulocyte precursor cells (CMGPs, Figure 6B). No differences were highlighted in the percentage number of the CMPs between WT and TNFAIP3^cx3cr1-KO^ mice (Figure 6A, Mann–Whitney test *p* = 0.073). Conversely, the analysis revealed a decrease of CMGPs in TNFAIP3^cx3cr1-KO^ mice compared to their WT littermates (Figure 6B, Mann–Whitney test *p* = 0.006).

**Figure 7:**
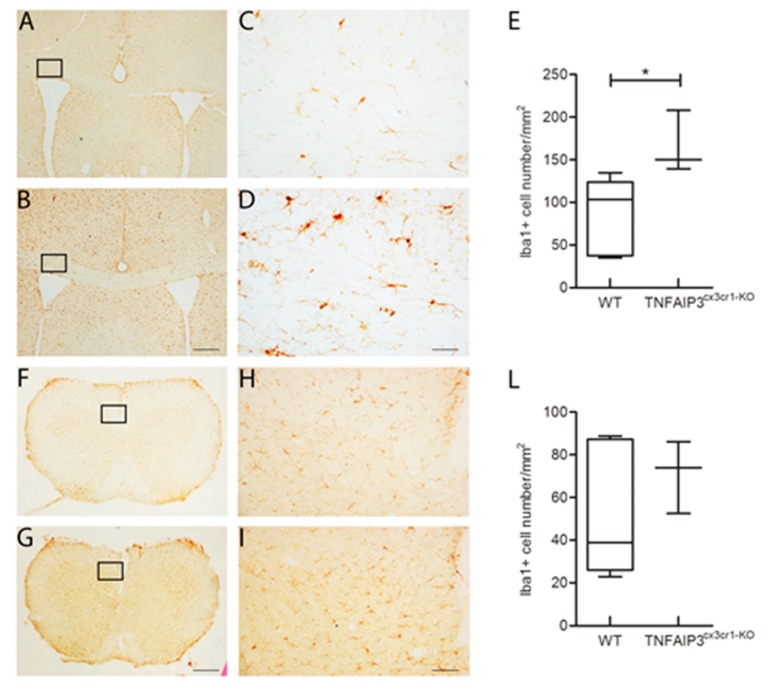
Ib1a+ microglial cells in the corpus callosum and in the spinal cord obtained from 3 month-old WT and TNFAIP3^cx3cr1-KO^ mice. (**A**–**D**; **F**–**I**) Representative images of coronal section of corpus callosum (**A**–**D**) and spinal cord (**F**–**I**) of WT (**A**,**C**,**F**,**H**) and TNFAIP3^cx3cr1-KO^ (**B**,**D**,**G**,**I**) mice immunostained with Iba1 antibody. Scale bar, 250 µm for A–B, F–G; 50 µm for C–D, H–I. (**E**,**L**) Quantitative analysis of the Iba1+ cell density in corpus callosum (**E**) and spinal cord (**L**) of WT and TNFAIP3^cx3cr1-KO^ mice discloses an increased microglial cell density in the corpus callosum of TNFAIP3^cx3cr1-KO^ mice compared to their WT littermates (**E**, *p* = 0.024). No differences are highlighted in the cervical spinal cord of TNFAIP3^cx3cr1-KO^ mice compared to their WT littermates (**L**, *p* = 0.548). (WT *n* = 6; TNFAIP3^cx3cr1-KO^ *n* = 3) (Mann–Whitney test, * *p* < 0.05). (Iba1, Ionized calcium binding adaptor molecule 1).

Considering that the TNFAIP3^cx3cr1-KO^ mice lead the absence of TNFAIP3 gene also in microglia and that the microglial phenotype has been already studied in the inducible TNFAIP3^cx3cr1-KO^ mice by Voet and colleagues [22], we confirmed their results on microglial cell density in the CNS parenchyma. Specifically, the immunohistochemistry analysis was performed in the corpus callosum and in the spinal cord of 3 month-old WT (*n* = 6) and TNFAIP3^cx3cr1-KO^ (*n* = 3) littermates. Representative images of coronal section of corpus callosum and spinal cord immunostained with Iba1 antibody were reported in Figure 7. The quantification analysis of the microglial cell density showed that TNFAIP3^cx3cr1-KO^ mice have an increased microglial cell density in the corpus callosum compared to their WT littermates (Figure 7A, Mann–Whitney test *p* = 0.024). No differences were detected in the microglial cell density of the spinal cord between WT and TNFAIP3^cx3cr1-KO^ mice (Figure 7B–D, Mann–Whitney test *p* = 0.548). These data indicate that the lack of TNFAIP3 influence also the myeloid glial compartment.

## 3. Discussion

TNFAIP3 is a central gatekeeper in inflammation able to inhibit the NF-kB pathway [3,4,5,6]. TNFAIP3 has been genetically associated with different inflammatory autoimmune diseases [7,8,9,10], among which MS [11]. We previously demonstrated a significant down-regulation of the TNFAIP3 transcript level in peripheral blood obtained from treatment-naïve MS patients in comparison to HC [12,13,14,15,23]. Further researches revealed that the unbalanced expression of TNFAIP3 in MS was also present in the monocyte cell population and not only on whole blood [16]. Interestingly, the analysis of the role of TNFAIP3 in myeloid cells deriving from studies in murine models is still incomplete. In fact, previous works induced the deficiency of TNFAIP3 in M-MDC [20] or in microglia [22], but never in the entire but ontogenetically distinct myeloid compartments. Here, we evaluated the effects of the TNFAIP3 lack in myeloid lineage, including M-MDC and microglia using a conditional KO murine model in which the CRE recombinase is controlled by the myeloid-specific cx3cr1 promoter element [24]. Three month-old TNFAIP3^cx3cr1-KO^ mice were vital and showed a macroscopic normal appearance. Notably, we did not observe in the TNFAIP3^cx3cr1-KO^ mice signs of paws inflammation, as reported in the conditional KO model in which the TNFAIP3 gene was deleted only in M-MDC and not in microglia [20]. On the other hands, our results are in agreement with the data obtained from Voet and colleagues in which the deficiency of TNFAIP3 in microglia is not related to alteration in gross anatomy of mice [22]. However, we observed a body weight reduction in TNFAIP3^cx3cr1-KO^ mice compared to their WT littermates. To date, no results related to the body weight in conditional TNFAIP3 KO mice are reported, but it has been demonstrated that the germinal deletion of TNFAIP3 induces cachexia [19]. Nevertheless, the molecular mechanism by which the lack of TNFAIP3 could affect body weight is not known. 

In order to detect the specific role of TNFAIP3 in myeloid cells, we performed flow-cytometry analysis on the lymphoid organs of the conditional TNFAIP3^cx3cr1-KO^ mice. We revealed, in agreement with previous studies, a decreased number of macrophages and monocytes in TNFAIP3^cx3cr1-KO^ mice in comparison to WT. These observations suggest that TNFAIP3 is physiologically involved in the development of myeloid lineage and therefore its absence could compromise its development. Notably, DCs and B cells are reduced in number in the spleen of TNFAIP3^cx3cr1-KO^ mice, but they are increased in lymph nodes in comparison to those of WT littermates. The opposite result is not completely understood but could be related to a different effect of TNFAIP3 deletion during distinct phases of their maturation that they encounter in the two compartments. However, further studies are necessary to dissect this behavior. Here, we also pointed out for the first time that myeloid TNFAIP3 lack induces a decrease in the amount of NK cells, which are potent cytotoxic killers belonging to the innate immune system. To date, no results related to NK cells in conditional TNFAIP3 KO mice are still reported. We also indicated that the amount of CD8+ T cytotoxic cells is increased in TNFAIP3^cx3cr1-KO^ mice in comparison to WT littermates. This datum is in agreement with a previous study reporting a higher percentage of these cells in the inguinal lymph nodes of myeloid-TNFAIP3 deficient mice [20]. However, although myeloid-TNFAIP3 deficiency increases the number of CD8+ T cells, the exact role of these T lymphocytes in autoimmune CNS inflammation remains controversial, supporting both pathogenic [25] and protective [26] roles in MS and EAE. Collectively, the de-regulated production of M-MDC due to the lack of TNFAIP3 could be related to general altered immune phenotype involving not only myeloid cells but also lymphocytes, such as B, T and NK cells.

Thanks to the analysis performed on the bone marrow of the conditional TNFAIP3^cx3cr1-KO^ mice, we highlighted for the first time that the immunological alteration did not occur only during the development of immune cells but was already present in the precursor cells. 

Related to the CNS parenchyma, we evaluated microglia in two regions represented by corpus callosum and spinal cord. We have chosen corpus callosum because it is the largest white matter structure in the brain and is often injured in MS patients [27]. The analysis in this region allows us to lay the foundations for understanding the consequence of TNFAIP3 deficiency in microglia during well-established demyelinating processes. In addition, to confirm the results of Voet and colleagues [22], we also included the analysis of the spinal cord that they had already evaluated. We observed an increased microglia cell density in the corpus callosum of TNFAIP3^cx3cr1-KO^. No differences were detected in the microglial cell density of spinal cord between WT and TNFAIP3^cx3cr1-KO^ mice probably due to the small sample size. The obtained results suggest that TNFAIP3 in microglia is needed to control neuroinflammation that is strictly connected with microglia proliferation. In particular, TNFAIP3 could physiologically limit the expansion of microglia to maintain CNS homeostasis and therefore its absence could contribute to neuroinflammatory events that occur in CNS pathology. However, considering the well dissected role of TNFAIP3 in microglia acting specifically on neuronal synaptic function, we decided to not deep insight in this aspect that it has been already fully dissected [22]. In conclusion, the obtained results support the suggested critical role of TNFAIP3 in the control of myeloid cells. In addition to its role in M-MDC, the myeloid-TNFAIP3 lack is related to the altered development of other immune cells, such as B, T and NK cells. Furthermore, in this study we corroborated previous work [22] reporting an increase in cell density of microglia in CNS parenchyma in TNFAIP3 deficiency condition suggesting that TNFAIP3 is necessary to control the glial neuroinflammatory processes. Collectively, this work even if performed on a small sample size, supports the already partially tested critical role of TNFAIP3 in controlling the development of M-MDC and microglia.

## 4. Materials and Methods 

### 4.1. Animals

All experimental procedures were carried out at the Neuroscience Institute Cavalieri Ottolenghi (NICO, Orbassano, Turin, Italy), approved by the Ethical Committee of the University of Torino and authorized by the Italian Ministry of Health (authorization number: 59/2016-PR). The experiments have been carried out in accordance with the European Communities Parliament and Council Directives of 24 November 1986 (86/609/EEC) and 22 September 2010 (2010/63/EU). Mice were housed with a 12 h light/dark cycle and free access to food/water. Adequate measures were taken to minimize pain and discomfort.

TNFAIP3^flox/flox^ mice not commercially available, were obtained from the no-profit RIKEN BioResource Center (Wakō, Saitama Prefecture, Japan). The mice were deposited from Dr. H. Honda (http://www.brc.riken.jp/inf/en/index.shtml, RBRC05494) [28]. The transgenic mice carrying the CRE recombinase under the control of the myeloid-specific chemokine C-X3-C motif receptor 1 (cx3cr1-CRE^+/-^) promoter element were purchase from The Jackson Laboratories (Bar Harbor, ME, US) (B6J.B6N(Cg)-cx3cr1^tm1.1(CRE)Jung^/J; stock No: 025524) [24]. Three month-old female and male TNFAIP3^flox/flox^::cx3cr1-CRE^+/-^ (TNFAIP3^cx3cr1-KO^, homozygous knock-out), and TNFAIP3^flox/flox^::cx3cr1-CRE^-/-^ (WT, wild-type) mice were included in each group and used for all experimental paradigms. Their genotype was confirmed by means of polymerase chain reaction (PCR) according to the manufacturer’s protocol [24,28].

### 4.2. Immunological Procedures

Three month-old TNFAIP3^cx3cr1-KO^ (*n* = 4) and WT (*n* = 8) littermate mice were euthanized by inhalation of isoflurane. Spleen, axillary and inguinal lymph nodes and femoral bone were removed and immersed in fresh phosphate buffered saline (PBS). To collect bone marrow, the femurs were cut at both ends and with a 23 G (gauge) needle and using a 10 mL syringe filled with ice-cold PBS, the bone marrow was flushed out. Tissues (i.e., spleen, axillary and inguinal lymph nodes) were manually homogenized with a syringe, and rinsed with PBS. The suspended cells obtained from spleen, lymph nodes and bone marrow were centrifuged at 1500 RPM for 10 min. The supernatant was discarded and the pellet was suspended in 3 mL of red blood cells lysis solution (Z3141, Promega, Madison, WI, US) and incubated at 4 °C for 10 min. After adding 10 mL of PBS, the cells were centrifuged at 1500 RPM for 10 min. The supernatant was discarded and the cells were cryopreserved at−80 °C until use in Roswell Park Memorial Institute (RPMI) 1640 (Invitrogen Life Technologies, Grand Island, NY, US), supplemented with 30% heat-inactivated fetal bovine serum (FBS, Invitrogen Life Technologies, Carlsbad, CA, US) and 10% dimethyl sulfoxide (DMSO, Sigma-Aldrich, St Louis, MO, US).

### 4.3. Flow-Cytometry 

After gentle thawing at 37 °C, the cryopreserved cells obtained from spleen, lymph nodes and bone marrow were immediately added to 5 mL RPMI 1640 (Invitrogen Life Technologies, Carlsbad, CA, US), supplemented with 10% heat-inactivated FBS (Invitrogen Life Technologies) and centrifuged to remove DMSO. Samples were re-suspended in RPMI 1640 medium supplemented with 10% heat-inactivated FBS and counted for flow-cytometry experiments. Macrophages (i.e., CD11b^+^F4/80^+^), monocytes (i.e., CD11b^+^Ly-6C^+^Ly-6G^+^), dendritic cells (DCs) (i.e., CD11c^+^CD86^+^), natural killer (NK) (i.e., CD49b^+^), NK T (i.e., CD3^+^CD49b^+^), T (i.e., CD3^+^, CD3^+^CD4^+^, CD3^+^CD8^+^) and B (i.e., CD45R-B220^+^) cells were evaluated in the same spleen and lymph nodes specimen of 3 month-old TNFAIP3^cx3cr1-KO^ and WT littermate mice. The common myeloid precursors (CMPs, Lin^-^Sca1^-^c-kit^+^CD34^+^CD16/CD32^-^) and the common monocyte and granulocyte precursors cells (CMGPs, Lin^-^Sca1^-^c-kit^+^CD34^+^CD16/CD32^+^) were evaluated in the same bone marrow specimen of 3 month-old TNFAIP3^cx3cr1-KO^ and WT littermate mice. Non-specific sites of 1 × 10^6^ cells were blocked with rabbit immunoglobulins G (IgG, Sigma-Aldrich, Saint Luis, MI, US), and 1 × 10^6^ cells were then incubated with fluorochrome-conjugated monoclonal Ab (mAb) and isotype-matched negative controls for 10 min at 4 °C.

The following anti-mouse mAbs were used: CD3-allophycocyanin (APC, 130-109-838), CD45R-B220-phycoerythrin (PE, 130-102-292), CD49b-fluorescein isothiocyanate (FITC, 130-102-258), CD11c-APC (130-102-493), F4/80-APC (130-102-379), CD86-PE (130-102-604), c-kit-(CD117)-PE (130-111-693), CD16/CD32-PE (130-102-429) (Miltenyi Biotec, Bergisch Gladbach, Germany) and CD4-phycoerythrin-cy7 (PECy7, BMS25-0041-82), CD8-fluorescein isothiocyanate (FITC, BMS11-0081-82), CD11b-PE (BMS12-0112-82), Ly6-C-AlexaFluo488 (BMS53-5932-82), Ly6-G-APC (BMS17-5931-82), Lineage cocktail (Lin)-PerCP-Cy5.5 (BD Pharmingen, 51-9006964, San Jose, CA, US), Sca1-APC (130-106-425), CD34-FITC (130-105-831) (eBioscience, ThermoFisher Scientific, San Diego, CA, US). Living cells identified by propidium iodide (Sigma-Aldrich) exclusion were gated according to their light-scatter properties to exclude cell debris. Samples were analyzed using a CyAn ADP, running Summit 4.3 software (Beckman Coulter, Brea, CA, US). The gating strategies are shown in the Figure 1 for spleen and lymph nodes and in Figure 2 for bone marrow.

### 4.4. Histological Procedures

Three month-old TNFAIP3^cx3cr1-KO^ (*n* = 3) and WT (*n* = 6) littermate mice were deeply anesthetized (ketamine 200 mg/kg, xylazine 50 mg/kg) and trans-cardially perfused with 4% paraformaldehyde in 0.12 M phosphate buffer, pH 7.2–7.4. Brain and spinal cord were removed and immersed in the same fixative at 4 °C for 24 h and then cryo-protected in 30% sucrose in 0.12 M phosphate buffer as reported in [29]. Brains and spinal cords were frozen and serially cut by a cryostat in 30 µm-thick coronal sections collected in PBS. In order to detect microglia, slices were incubated overnight at 4 °C with the polyclonal anti-rabbit Ionized Calcium Binding Adaptor molecule 1 (Iba1, 019-19741, WAKO, Japan) diluted 1:1000 in PBS with 0.25% Triton X-100 and 1.5% normal donkey serum (NDS). The secondary biotinylated anti-rabbit antibody (715 065 152, Jakson Immuno Research, Philadelphia, PA, US) diluted 1:100 in a solution of PBS containing 0.2% Triton-X100 and 1.5% of NDS were used. Immunohistochemical reactions were performed with the avidin–biotin–peroxidase method (Vectastain ABC Elite kit; Vector Laboratories, Burlingame, CA, US) and revealed using 3,3’-diaminobenzidine (3% in Tris–HCl) as chromogen. Images for morphometric analysis were acquired by means of the Nikon Eclipse E600 microscope and analyzed by means of the Cell Counter Tools of ImageJ software (http://rsbweb.nih.gov/ij/index.html). Iba1 positive (+) cell number (cell number/mm^2^) in the corpus callosum (Bregma 1.10 mm, Interaural 4.90; Bregma -0.82 mm, Interaural 2.98) and in spinal cord section at cervical level were quantified. At least 3 sections for each animal were evaluated.

### 4.5. Statistical Analysis

Statistical analysis was performed using GraphPad Prism (GraphPad Software, version 5; San Diego, CA, US). Continuous data were presented as medians and ranges and categorical data were given as counts and percentages. Normality of distribution was assessed by the Shapiro–Wilk test. The differences between groups were calculated with Mann–Whitney U test. *p*-values < 0.05 were considered significant. 

## 5. Conclusions and Future Prospects

In agreement with previous published results, we conclude that TNFAIP3 is involved in the development of myeloid cells not only in blood but also in brain parenchyma. Notably, the absence of TNFAIP3 in myeloid cells also alters the homeostasis of lymphoid division. Considering the role that M-MDC and microglia exert during the pathogenesis of autoimmune inflammatory diseases, such as MS, the knowledge in this field of research could lead to the identification of novel therapeutic strategy for these disorders that are still untreated.

## Supplementary Materials

Supplementary materials can be found at https://www.mdpi.com/1422-0067/21/8/2830/s1.
