# Supplementary material for: TNFAIP3 Deficiency Affects Monocytes, Monocytes-Derived Cells and Microglia in Mice
Source: Int J Mol Sci. 2020 Apr 18;21(8):2830. doi: 10.3390/ijms21082830 (PMC7215837; doi:10.3390/ijms21082830)
Supplement: Supplementary file 1 [file ijms-21-02830-s001.pdf]

Table 1S. Summary table of the flow cytometry results obtained in spleen, lymph nodes and bone marrow from 3 months-old WT and TNFAIP3$^{cx3cr1\text{-}KO}$ mice

	Spleen	Lymph nodes	Bone marrow
	**TNFAIP3$^{cx3cr1\text{-}KO}$ versus WT**	**TNFAIP3$^{cx3cr1\text{-}KO}$ versus WT**	**TNFAIP3$^{cx3cr1\text{-}KO}$ versus WT**
**CD11b$^+$F4/80$^+$ macrophages**	↓↓	↓↓	not analyzed
**CD11b$^+$Ly6-C$^+$Ly6-G$^+$ monocytes**	↓	NC	not analyzed
**CD11c$^+$CD86$^+$ dendritic cells**	↓	↑	not analyzed
**CD49b$^+$ NK-cells**	NC	↓↓	not analyzed
**CD3$^+$CD49b$^+$ NK T-cells**	NC	↓↓	not analyzed
**B220$^+$ B-cells**	↓	↑	not analyzed
**CD3$^+$ T-cells**	NC	NC	not analyzed
**CD3$^+$CD4$^+$ T-cells**	NC	NC	not analyzed
**CD3$^+$CD8$^+$ T-cells**	NC	↑	not analyzed
**Common myeloid precursors (CMPs)**	not analyzed	not analyzed	NC
**Common monocyte and granulocyte precursor cells (CMGPs)**	not analyzed	not analyzed	↓↓

↓↓; percentage number of cells is significantly reduced in TNFAIP3$^{cx3cr1\text{-}KO}$ compared to WT mice (Mann-Whitney test, ** p<0,01)

↓; percentage number of cells is significantly reduced in TNFAIP3$^{cx3cr1\text{-}KO}$ compared to WT mice (Mann-Whitney test, * p<0,05)

↑; percentage number of cells is significantly increased in TNFAIP3$^{cx3cr1\text{-}KO}$ compared to WT mice (Mann-Whitney test, * p<0,05)

NC; no changes observed
